# Perioperative strategy in colonic surgery; *LA*paroscopy and/or *FA*st track multimodal management versus standard care (LAFA trial)

**DOI:** 10.1186/1471-2482-6-16

**Published:** 2006-11-29

**Authors:** Jan Wind, Jan Hofland, Benedikt Preckel, Markus W Hollmann, Patrick MM Bossuyt, Dirk J Gouma, Mark I van Berge Henegouwen, Jan Willem Fuhring, Cornelis HC Dejong, Ronald M van Dam, Miguel A Cuesta, Astrid Noordhuis, Dick de Jong, Edith van Zalingen, Alexander F Engel, T Hauwy Goei, I Erica de Stoppelaar, Willem F van Tets, Bart A van Wagensveld, Annemiek Swart, Maarten JLJ van den Elsen, Michael F Gerhards, Laurens Th de Wit, Muriel AM Siepel, Anna AW van Geloven, Jan-Willem Juttmann, Wilfred Clevers, Willem A Bemelman

**Affiliations:** 1 Department of Surgery, Academic Medical Center Amsterdam, The Netherlands; 2 Department of Anesthesiology, Academic Medical Center Amsterdam, The Netherlands; 3 Department of clinical epidemiology and biostatistics, Academic Medical Center Amsterdam, The Netherlands; 4 Department of Surgery, University Hospital Maastricht, The Netherlands; 5 Department of Surgery, VU Medical Center Amsterdam, The Netherlands; 6 Department of Anesthesiology, VU Medical Center Amsterdam, The Netherlands; 7 Department of Surgery, Zaans Medical Center Zaandam, The Netherlands; 8 Department of Anesthesiology, Zaans Medical Center Zaandam, The Netherlands; 9 Department of Surgery, Sint Lucas Andreas Hospital Amsterdam, The Netherlands; 10 Department of Anesthesiology, Sint Lucas Andreas Hospital Amsterdam, The Netherlands; 11 Department of Surgery, Onze Lieve Vrouwen Gasthuis Amsterdam, The Netherlands; 12 Department of Anesthesiology, Onze Lieve Vrouwen Gasthuis Amsterdam, The Netherlands; 13 Department of Surgery, Hilversum Hospital, The Netherlands; 14 Department of Anesthesiology, Hilversum Hospital, The Netherlands

## Abstract

**Background:**

Recent developments in large bowel surgery are the introduction of laparoscopic surgery and the implementation of multimodal fast track recovery programs. Both focus on a faster recovery and shorter hospital stay.

The randomized controlled multicenter LAFA-trial (LAparoscopy and/or FAst track multimodal management versus standard care) was conceived to determine whether laparoscopic surgery, fast track perioperative care or a combination of both is to be preferred over open surgery with standard care in patients having segmental colectomy for malignant disease.

**Methods/design:**

The LAFA-trial is a double blinded, multicenter trial with a 2 × 2 balanced factorial design. Patients eligible for segmental colectomy for malignant colorectal disease *i.e*. right and left colectomy and anterior resection will be randomized to either open or laparoscopic colectomy, and to either standard care or the fast track program. This factorial design produces four treatment groups; open colectomy with standard care (a), open colectomy with fast track program (b), laparoscopic colectomy with standard care (c), and laparoscopic surgery with fast track program (d). Primary outcome parameter is postoperative hospital length of stay including readmission within 30 days. Secondary outcome parameters are quality of life two and four weeks after surgery, overall hospital costs, morbidity, patient satisfaction and readmission rate.

Based on a mean postoperative hospital stay of 9 +/- 2.5 days a group size of 400 patients (100 each arm) can reliably detect a *minimum *difference of 1 day between the four arms (alfa = 0.95, beta = 0.8). With 100 patients in each arm a difference of 10% in subscales of the Short Form 36 (SF-36) questionnaire and social functioning can be detected.

**Discussion:**

The LAFA-trial is a randomized controlled multicenter trial that will provide evidence on the merits of fast track perioperative care and laparoscopic colorectal surgery in patients having segmental colectomy for malignant disease.

## Background

Recent developments in large bowel surgery are the introduction of laparoscopic surgery and the implementation of multimodal fast track perioperative care programs. Both focus on enhanced recovery and shorter hospital stay as compared to open surgery and traditional care. Laparoscopic colectomy was first described in 1991[1]. Since then a lot of effort has been made to establish its feasibility and safety particularly in laparoscopic colectomy for cancer. Recently, several randomized trials comparing laparoscopic with open colectomy indicated that laparoscopic surgery can be applied safely both for malignant and benign diseases [2-7]. Several systematic reviews that assessed the evidence on the laparoscopic approach for colorectal cancer reported that laparoscopic surgery, in a traditional perioperative care setting was associated with less morbidity, less postoperative pain, earlier recovery and shorter hospital stay[2,8,9]. Furthermore, short term cancer related outcomes such as cancer free resection margins and the number of harvested lymph nodes, as well as long term cancer related outcomes such as disease free survival were comparable between laparoscopic and open surgery[2]. These results stimulated many surgeons in the Netherlands to set up a laparoscopic colorectal program.

At the same time, enthusiasm was raised for the so-called fast track perioperative care program, also referred to as Enhanced Recovery After Surgery (ERAS^®^), which essentially is a modification of the program initially developed by the Danish surgeon Henrik Kehlet [10-13]. This multimodal program, involving optimalization of several aspects of the perioperative management of patients undergoing colectomy, enables patients to recover earlier and therefore go home as early as three days after open colectomy. Furthermore, postoperative morbidity was reduced [14-19]. The essence of a fast track perioperative care program consists of extensive preoperative counseling, no bowel preparation, no sedative premedication, no preoperative fasting but carbohydrate loaded liquids until two hours prior to surgery, tailored anesthesiology encompassing thoracic epidural anesthesia and short acting anesthetics, perioperative intravenous fluid restriction, minimally invasive surgery (*i.e*. through small incisions or laparoscopy), non-opioid pain management, no routine use of drains and nasogastric tubes, early removal of bladder catheter, standard laxatives and prokinetics, and early and enhanced postoperative feeding and mobilization [10-19].

As these new developments have been introduced in clinical practice, time has come to evaluate their feasibility, safety, and cost-effectiveness in large bowel surgery in a randomized controlled setting. It can be hypothesized that fast track and/or laparoscopy are associated with less attenuation of the patient's condition after surgery resulting in a shorter postoperative hospital stay, a faster recovery to full activity at home, and a better quality of life.

Since it has not been established which combination of perioperative management and surgical approach *i.e*. standard care, fast track care, laparoscopic surgery or open surgery is best in terms of postoperative hospital stay, quality of life, postoperative morbidity, readmission rate, overall costs and patient satisfaction, this is the subject of the present study proposal.

## Methods/design

### Study objectives

The objective of this study is to determine whether laparoscopic surgery, fast track perioperative care or a combination of both is to be preferred over open surgery with standard care in patients undergoing segmental colectomy for malignant disease. The objective is subdivided in three research questions; first, how laparoscopic surgery compares to open surgery in terms of hospital stay, quality of life and costs? Second, how fast track perioperative care compares to standard care in terms of hospital stay, quality of life, and costs? Finally, what is the added benefit of fast track perioperative care program in laparoscopic surgery in terms of hospital stay, quality of life and costs?

### Study design

**Figure 1:**
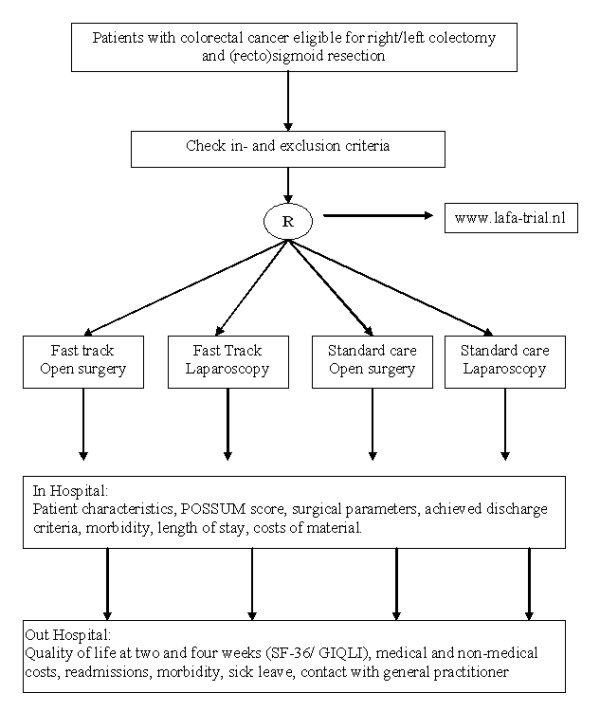
**LAFA-study Flowchart**. R = randomization, SF-36 = Short Form 36, GIQLI = Gastro-Intestinal Quality of Life Index, POSSUM score = Physiological and Operative Severity Score for the enUmeration of Mortality and Morbidity.

The LAFA-trial is a randomized multicenter trial, designed as a 2 × 2 balanced factorial design. Patients are blinded for the type of intervention *i.e*. laparoscopic or open surgery. Patients eligible for segmental colectomy, for malignant colorectal disease *i.e*. right and left colectomy and anterior resection will be randomized to either open or laparoscopic colectomy, and to either standard care or the fast track program. This factorial design results in four treatment groups; open colectomy with standard care (a), open colectomy with fast track perioperative care (b), laparoscopic colectomy with standard care (c), and laparoscopic surgery with fast track perioperative care (d) (see Figure 1).

Randomization is performed by an Internet randomization module. Block-randomization is used and the randomization is stratified for the randomizing centers.

### Primary and secondary endpoint

The primary endpoint of the LAFA-study is total postoperative hospital stay in days, including hospital stay of patients who are readmitted within 30 days after surgery.

Secondary endpoints are quality of life at two and four weeks after surgery. Quality of life will be measured by two validated questionnaires; Short Form 36 (SF-36) and the Gastro-Intestinal Quality of Life Index (GIQLI). Further secondary endpoints are; medical and non medical costs, morbidity, and mortality within 30 days after surgery, patient satisfaction measured by standardized questionnaires, and readmission rate.

### Participating centers

Seven Dutch hospitals of the LAFA-study group, including three academic centers and four non-academic centers, will enroll patients.

### Study population

The study population consists of patients eligible for segmental colectomy for malignant colorectal disease viz. right and left colectomy and anterior resection.

Inclusion criteria are; age between 40 and 80 years, colorectal cancer including colon and recto sigmoid cancers, ASA I-III, and informed consent.

Exclusion criteria are; prior midline laparotomy, ASA IV, laparoscopic surgeon not available, emergency surgery and a planned stoma.

### Ethics

This study is conducted in accordance with the principles of the Declaration of Helsinki and 'good clinical practice' guidelines. The independent medical ethics committees of the participating hospitals have approved the study protocol. Prior to randomization, written informed consent will be obtained from all patients.

### Study outline

Informed consent will be obtained at the outpatient department if the patient fulfills the inclusion and exclusion criteria. Randomization is performed instantly through the study website.

The randomization produces four treatment groups; open colectomy with standard care (a), open colectomy with fast track perioperative care (b), laparoscopic colectomy with standard care (c), and laparoscopic surgery with fast track perioperative care (d) (see Figure 1).

Patients that are randomized to fast track perioperative care will be informed by a "fast track" trial nurse and by the anesthesiologist about the essence of the fast track program. Appointments for these consultations will be made after consulting the surgeon and randomization has been done. All patients randomized to have a fast track perioperative treatment will be admitted to a separate "fast track" ward, where the nurses and medical staff are trained in fast track perioperative management.

Patients who will receive standard treatment are not counseled by the fast track nurse and will have a standard preassessment by the anesthesiologist.

Patient and medical staff will be blinded for the surgical approach until the day of discharge by applying a covering abdominal bandage.

#### Surgery

Both open and laparoscopic surgery is done according to the technique applied by the local surgeon. Antibiotic prophylaxis is done according to hospital protocol. All patients will have two enemas before surgery (evening before and morning before). After surgery the surgical wounds are covered with a abdominal dressing in order to blind the medical staff for the type of approach. A requirement for the participating laparoscopic surgeons to perform laparoscopic colectomy for cancer is a minimum of 20 laparoscopic colectomies for benign disease as indicated by the proclamation of the American Society of the Colon and Rectum Surgeons in 2004[20,21].

#### Fast track and standard care

Comparison of the different strategies is only possible when a fast track program is running sufficiently and patients are nursed separately depending on the results of randomization either on a standard care or fast track ward in order to avoid a bias towards fast track treatment by the nursing and medical staff. Patients that have standard care cannot be nursed by nurses that have experience with fast track care. Fast track multimodal management is done according to the protocol summarized in Table 1.

#### Discharge criteria

Since hospital stay is a primary efficacy parameter, the discharge criteria are defined. Every postoperative day will be noted whether the discharge criteria are met, and other reasons of prolonged hospital stay *e.g*. social environment or patient in acceptance. The discharge criteria include adequate pain control with oral analgesics, no nausea, ability to take solid foods, passage of flatus and/or stool, mobilization and self support as compared to the preoperative level, and acceptance by the patient.

### Statistical analysis

#### Intention to treat

The analysis will be performed in accordance with the intention to treat principle.

#### Sample size calculation

Since both, fast track care and laparoscopy focus on earlier recovery resulting in a reduction of hospital stay, the latter is used as primary efficacy parameter. The mean postoperative hospital stay for segmental bowel resection with standard care is 9 days with a standard deviation of 2.5 days in the Academic Medical Center Amsterdam. Using a 5% significance level, a total sample size of 400 would have a power of >95% to detect a minimum reduction of 1 day in hospital stay between laparoscopic surgery and open surgery, a 1 day reduction in hospital stay between fast track care and standard care, and a power of 80% to detect the same difference between the combination of fast track care with laparoscopic surgery and current treatment.

A much larger difference can be expected between the treatment groups, for instance open surgery and standard care compared to fast track perioperative care and laparoscopic surgery. In order to obtain results with adequate precision we have calculated group size using a difference of 1 day rather than the expected 2–4 days. With a group size of one hundred patients per arm it is possible to find a significant difference (alfa = 0.05, beta = 0.1) of at least 10% in subscales of the SF-36, a validated Quality of life Questionnaire, at two weeks after surgery [22-24]. Liem *et al*. demonstrated 20–30% differences in subscales of the SF-36 between laparoscopic versus open hernia repair 1 week after surgery[24]. Maartense *et al*. found a 10% difference in physical and social function two weeks after surgery comparing laparoscopic versus open ileocolic resection in a randomized study from our institution[25].

#### Economic evaluation

The marginal direct medical, non-medical and time cost differences will be calculated for the four treatment strategies. These will include the additional costs of laparoscopy, of fast track care, as well as the differences due to complications and readmissions.

### Data collection and monitoring

Data are collected via a secured Internet module which is specially designed for the LAFA-study. Data are collected daily until the day of discharge. Preoperatively, and at two and four weeks postoperatively the questionnaires (SF-36/GIQLI) are filled in by the patient. One month postoperatively, the general practitioner is contacted to inform whether he/she was contacted by the patient for problems related to the operation.

There will be regular contact between the study coordinators and the participating centers. One research fellow will monitor the included data of every patient.

## Discussion

Fast track programs in colonic surgery have been introduced more than a decade ago with favorable early results. Many elements of these fast track programs are based on solid evidence derived from randomized trials and systematic reviews. However, it is quite surprising, that implementation in daily practice has so far stayed behind [26-28] This can partly be explained by the necessity to break with long-standing traditions, such as preoperative fasting, slow postoperative advancement of oral feeding, and delayed mobilization. In a recent systematic review including six comparative single centre studies, fast track programs were found to reduce the time spent in the hospital and were found to be safe in major abdominal surgery. However, this systematic review demonstrated that the evidence on fast track colonic surgery was scarce[29].

Both, laparoscopic surgery and fast track programs are costly and require extensive expertise. Laparoscopic surgery is costly due to expensive disposables and additional operating time. Furthermore, a considerable learning curve must be mastered. Only 5–8% of the colectomies are therefore done laparoscopically in the Netherlands. Fast track multimodal perioperative care requires additional personnel trained in several aspects of the fast track program to make the program work. It is clear that both, laparoscopic surgery and fast track programs enhance recovery and thereby reduce hospital stay[2,8,29-32]. Shortening hospital stay and reduction of morbidity are attractive, since both increase the availability of beds and might reduce the overall cost of hospital stay. However, despite the current enthusiasm and implementation into daily practice of fast track care and laparoscopic surgery, there are few data available that provide evidence on the optimal combination (laparoscopic or open surgery and fast track or standard care) in terms of shorter hospital stay, reduced morbidity and cost effectiveness.

The largest reduction in hospital stay can probably be achieved by a combination of fast track programs and laparoscopic surgery. However, it is not known what the additional costs of laparoscopic surgery or fast track programs are compared to the reduction in hospital stay that can be achieved with these programs. Since the average postoperative hospital stay after segmental colectomy is still considerable in the Netherlands as well as throughout Europe, an enormous improvement can be expected applying fast track programs and/or laparoscopy. What the relative contribution is in reduction in hospital stay of both methods is unknown. This must be assessed in a setting where patients are blinded for the approach of surgery. The randomized controlled LAFA-trial was conceived to determine whether laparoscopic surgery, fast track perioperative care or a combination of both is to be preferred over open surgery with standard care in patients undergoing segmental colectomy for malignant disease.

## Abbreviations

LAFA-trial: LAparoscopy and/or Fast track multimodal management versus standard care trial

ASA: American Society of Anaesthesiologists

ERAS^®^: Enhanced Recovery After Surgery

GIQLI: Gastro-Intestinal Quality of Life Index

SF-36: Short Form-36

## Competing interests

The author(s) declare that they have no competing interests.

## Authors' contributions

JW drafted the manuscript. WAB co-authored the writing of the manuscript. All other authors participated in the design of the study during several meetings and are local investigators at the participating centers.

All authors edited the manuscript and read and approved the final manuscript.

## Pre-publication history

The pre-publication history for this paper can be accessed here:



## Figures and Tables

**Table 1:** The essence of the fast track care program and standard care program.

	***FAST-TRACK CARE***	***STANDARD CARE***
***PRE-OPERATIVE PHASE***		
*Outpatient department of Surgery*	- Scheduling of operation -Information about the fast track program -Informed consent	- Scheduling of operation -Informed consent
*Outpatient department of anesthesiology*	- Pre-assessment for risk adjustment -Discussion focusing on placement of thoracic epidural catheter for management of perioperative analgesia -Discussion of the essence of the fast track program	- Pre-assessment for risk adjustment -Open discussion about different possibilities for management of perioperative analgesia
*Pre-admission counseling and guided tour on surgical ward*	- Yes	- No
***DAY OF ADMISSION***		
*Intake*	- Additional fast track information	- Routine
*Bowel preparation*	- Only enema	- Only enema
*Pre-operative carbohydrate loaded liquids*	- 4 units (preOp^®^)	- No
*Diet*	- Last meal 6 h before operation	- Last meal until midnight
*Pre-anesthetic evening medication*	- Lorazepam, 1 mg the evening before operation, *if necessary*	- Lorazepam, 1 mg or Temazepam 10 or 20 mg
***DAY OF SURGERY***		
*Pre-operative fasting*	- No, 2 units CHL 2 h before surgery	- Yes
*Pre-anesthetic medication*	- No	- Lorazepam 1 mg, or Midazolam 7.5 mg
*Anesthetic management*	- Placement of thoracic epidural catheter (T6–T10, depending on the surgical resection); *test-dose *(Bupivacaine 0.25% with adrenaline 1:200,000), *top-up dose *(Bupivacaine 0.25% [± 10 ml] with Sufentanil 25 μg, followed by *continuous infusion *(Bupivacaine 0.125% with Fentanyl 2.5 μg.ml^-1^) until day 2 postoperative -Combined with balanced general anesthesia -Restricted per-operative fluid infusion regime (Ringers lactate 20 ml.kg^-1 ^in the 1^st ^h followed by 6 ml.kg^-1^.h^-1^) -Use of vasopressor drugs as 1^st ^choice for management of mean blood pressure drop > 20% of baseline -Forced body heating (Bair hugger system and warmed IV fluids) -Removal of naso-gastric tube before extubation -Prophylactic use of Odansetron (4 mg) to prevent PONV	- Placement of thoracic epidural conform fast track group, or lower level, or PCA-pump. -Combined with balanced general anesthesia -Standard per-operative fluid infusion regime (Ringers lactate 20 ml.kg^-1 ^in the 1^st ^h followed by 10–12 ml.kg^-1^.h^-1^) -Use of extra fluid challenge as 1^st ^choice for management of mean blood pressure drop > 20% below baseline -Forced body heating (Bair hugger system and warmed IV fluids) -Removal of naso-gastric tube before extubation -Use of Odansetron, Dexamethason or Droperidol for PONV management according to attending anesthesiologist
*Surgical Management*	- Minimal invasive incisions/laparoscopy -Supra-pubic urine catheter -Infiltration of surgical wounds with Bupivacaine -No standard use of abdominal drains	- Median laparotomy approach/laparoscopy -Urine catheter according to attending surgeon -No infiltration of surgical wounds with local anesthetic drugs -No standard use of abdominal drains
*Early post-operative management*	- Use of epidural catheter as mentioned before to which Paracetamol 4 × 1 g.d^-1 ^is added -First oral drinks at 2 h post-surgery, supplemented with CHL liquids, 2 units (Nutridrink^®^) -IV infusion of Ringers lactate 1.5 l.d^-1^ -Mobilization in the evening (>2 h out of bed) -First semi-solid food intake in the evening	- Epidural or PCA-morphine to which Paracetamol 4 × 1 g.d^-1 ^and/or Diclofenac 3 × 50 mg.d^-1 ^are added -Small amount of water orally -IV infusion of Ringers lactate 2.5 l.d^-1^ -No mobilization scheme
***DAY 1 AFTER SURGERY***		
*Postoperative Management*	- Oral intake > 2 l (including 4 units CHL liquids) -Normal diet -Stop IV fluid administration (leave canulla) -Start laxative (MgO, 2 × 1 g.d^-1^) -Close supra-pubic urine catheter and remove when residue < 50 ml -Expand mobilization (> 6 h out of bed)	- Diet increased on daily basis -IV fluid administration (2.5 l.d^-1^) is continued till adequate oral fluid intake -Mobilization according to attending surgeon
**DAY 2 AFTER SURGERY**		
*Postoperative Management*	- Remove epidural add Diclofenac 3 × 50 mg.d^-1^ -Remove IV cannula -Continue Paracetamol 4 × 1000 mg and laxative -Normal diet -Expand mobilization (> 8 hours) -Plan discharge	- Epidural removed according to attending anesthesiologist -Continue as on day 1 untill discharge criteria are fulfilled
DAY 3 AFTER SURGERY	- Continue as on day 2 untill discharge criteria are fulfilled	Continue as on day 2 untill discharge criteria are fulfilled

CHL: CarboHydrate Loaded, PCA: Patient Controlled Anesthetics, IV: Intra Venous, PONV: PostOperative Nauseaand Vomiting, MgO: Magnesium Oxide
